# Individual and community-level factors associated with khat (*Catha edulis*) use among women of reproductive age in Halaba zone, South Ethiopia: a multilevel mixed effect analysis

**DOI:** 10.3389/fpsyt.2024.1333556

**Published:** 2024-02-19

**Authors:** Biruk Wogayehu, Tsegaye Demissie, Eskinder Wolka, Mekuriaw Alemayehu, Kassa Daka

**Affiliations:** ^1^ Department of Public Health, Arbaminch College of Health Sciences, Arbaminch, South Region, Ethiopia; ^2^ Department of Public Health, School of Public Health, College of Medicine and Health Sciences, Wolaita Sodo University, Sodo, South Region, Ethiopia; ^3^ Department of Epidemiology and Biostatistics, Institute of Public Health, College of Medicine and Health Sciences, University of Gondar, Gondar, Amhara Region, Ethiopia

**Keywords:** khat, *Catha edulis*, women of reproductive age, multilevel-analysis, Ethiopia

## Abstract

**Introduction:**

There is a paucity of data on factors associated with khat chewing among women of reproductive age using multilevel analysis. Furthermore, the effects of some potential factors like stressful life events, knowledge about and attitude toward the effects of khat have been given little attention and are not well understood. Therefore, this study aimed to examine the prevalence and multilevel factors associated with khat use among women of reproductive age in Halaba zone, South Ethiopia.

**Methods:**

A community-based cross sectional study was conducted in Halaba zone from February to July, 2023. Systematic random sampling technique was used to include 1573 study participants. The dependent variable was current khat use, which is operationalized as using khat within 30 days preceding the study. An interviewer administered questionnaire was used for the data collection.

**Results:**

The prevalence of current khat use among women of reproductive age was 65.9% [95%CI (63.5-68.2%)]. Factors significantly associated with khat use were; ages of women 35 and above years [Adjusted Odds Ratio (AOR) = 6.35, 95% CI: (3.62, 11.13)], ever married [AOR = 2.41, 95% CI: (1.10, 5.31)], secondary and above education [AOR = 0.28, 95% CI: (0.15, 0.49)], belong to richer household [AOR = 1.75, 95% CI: (1.12, 2.75)], mass media use [AOR = 3.12, 95% CI: (1.85, 4.81)], low knowledge about khat effects [AOR = 3.12, 95% CI: (1.85, 5.24)], positive attitude towards khat use [AOR = 11.55, 95% CI: (6.76, 19.71)], and strong social support [AOR = 0.43, 95% CI: (0.28, 0.64)] and non-user friend [AOR = 0.31, 95% CI: (0.20, 0.48)]. From the community level variables: rural residence [AOR = 5.06, 95% CI: (1.82, 14.09)] was significantly associated with khat use.

**Conclusion:**

Khat use among women of reproductive age was found to be very high. From individual-level factors: advanced ages of women, secondary and above education, live in the richer wealth quintile, mass media exposure, low knowledge on khat effects, positive attitude towards khat use, strong social support, and from community-level variables: residing in rural area were significantly associated with khat use. Khat use screening for all women of childbearing age, as well as referral to substance use disorder centers for those women identified as having khat use disorder, should become a standard of care in all health facilities.

## Introduction

### Background and significance

Khat plant *(Catha edulis)* is an evergreen tree of the Celastraceae family that is mainly cultivated for its stimulant leaves in East Africa and southwest Arabia. Khat grows at high altitudes of 1500–2500 meters. The khat tree needs nearly 10 years to reach maturity, but the shoots and leaves are harvested after 3 to 4 years (1, 2). According to historical records, khat usage began in ancient Ethiopia (Abyssinia) in the 13^th^ century, and khat leaves were introduced in Yemen in the first half of the 15^th^ century (3). The objective of this study is to examine the individual and community-level factors associated with khat use among women of reproductive age in Halaba Zone, South Ethiopia.

Understanding individual and community-level factors will assist planners, decision-makers, and policymakers in having a good understanding of the factors influencing khat use among reproductive-age women and taking appropriate measures to address khat-related social and health problems. Furthermore, identifying community-level characteristics related to khat use provides a full picture of the dimensions of khat usage, provides significant information to researchers, and highlights a clear gap for future studies.

### Chemical composition of khat

Khat contains numerous alkaloids and also over 40 different chemical compounds (4). About 90% of the active chemicals present in khat leaves are released during chewing. The peripheral and central stimulant alkaloid cathinone (S-(-)-a-aminopropiophenone) is considered to be the primary psychoactive compound in the khat leaves (5). However, cathinone is comparatively unstable and decomposes after harvesting into norephedrine and cathine. When the leaves are dried, the process is accelerated. As a result, only freshly picked khat leaves are fully efficacious (6, 7). Fresh khat contains 36-343 mg of cathinone on average (8, 9), as well as 83-120 mg of cathine and 8-47 mg of norephedrine per 100 g of leaves (8). Pharmacologically just like amphetamine and noradrenaline, with the ability to affect both the central and peripheral nervous systems (10).

### Psychoactive effects

The effects of khat on the chewer include an increase in energy, excitement, and sociability (11), followed by anxiety, irritability, and sleep problems (12). The stimulatory effects of khat appear about 30 minutes after the initiation of chewing khat and the effect may persist for 3 hours (13).

### Legal status of khat

Globally, there is inconsistency in the law regarding khat use. In 1950s, the WHO first conducted research into the health implications and pharmacology of khat. The findings in 1964 led to the United Nation Commission of Narcotic Drugs ruling against the need for universal legislation, leaving it to individual nations to choose whether health advice have to be given to users (14). Cathinone has been classified as a Schedule I substance under the Controlled Substances Act since 1993, whereas cathine was classified as a Schedule IV substance in 1988 (9). Because khat is not on the list of controlled substances, the legal status of the plant itself is frequently challenging and varies across countries (15). Many countries in North America, Europe and Asia currently have control over khat (15). In Yemen, khat is legal; however in neighboring Saudi Arabia, khat is banned (16). However, it remains legal in the majority of East African countries (9). In Ethiopia, there is no clear legal restriction on khat use in Ethiopia (17). Khat cultivation and consumption are spreading at an astonishing rate in Ethiopia (15). Khat has become the highest earning commodity next to coffee. Over the past twenty years, the amount of land dedicated to khat cultivation has grown by 160%, with hundreds of millions of kilograms produced each year (15).

### Health effects

Long-term khat use is usually accompanied by psychological, cardiac, neurological, and digestive health problems (9). Khat use results in an increase in heart rate and blood pressure in humans since cathinone’s indirect sympathomimetic action, which lasts for about 3-4 hours after use (18, 19). A cross-sectional study conducted in Ethiopia found that individuals who chew and smoke khat have significantly higher diastolic blood pressure (20). There is also evidence of an increased risk of cardiac arrhythmias and myocardial infarction (MI) (21, 22). It has been established that chewing khat has a detrimental impact on oral and dental tissues. These include keratotic lesions (23), mucosal pigmentation (24), temporomandibular joint problems (25), plasma cell gingivitis (25), tooth discoloration and attrition (25), and periodontal diseases (25).

Tannin astringency in khat leaves can cause oral mucosal keratosis, oesophagitis, and gastritis. According to reports, approximately 50% of khat chewers develop oral mucosal keratosis (26), which can progress to oral cancer (27). Khat chewing results in a weak flow of urination (28). The autonomic effect of cathinone on the peripheral nervous system was suggested to explain urine retention (29).

### Mental health disorders

Several studies have been conducted to investigate the potential link between excessive khat use and the risk of mental health disorders such as psychosis (30–34), depression, stress, anxiety (34, 35), mental distress (36), suicidal ideation (37), and paranoid ideation (38). A systematic review and meta-analysis of 35 studies found that the odds of psychotic symptoms among khat users were 2.22 times greater than the odds among non-khat users (39). In the same way, a meta-analysis of six studies conducted among Ethiopian college students found that students who used khat were 2.01 times more likely to experience common mental disorders than those who did not (40). Khat chewing has been linked to sleep problems as a result of a pattern of heavy use (41). People who chew khat complain about negative affects with greater frequency than non-chewers (42).

### Risky sexual behavior and khat consumption

Few studies in Africa have looked into the relationship between risky sexual behavior and khat consumption among women. A recent cross-sectional research conducted in Kampala, Uganda, showed that young substance consumers who had consumed khat in the last month had 93% higher odds of participating in multiple sexual partnerships in comparison to those who had not chewed (43). A study among female youth in the Amhara region, North Ethiopia revealed that khat consumers (p<0.01) were three times more likely to have risky sexual behavior as compared to non-user counterparts (44). In a similar manner, a cross-sectional study conducted among female high school students in eastern Ethiopia found that the use of khat was associated with higher levels of sexual violence victimization (45). As a result, the habit of khat chewing could fuel spread of sexually transmitted infections (46, 47).

### Khat use during pregnancy

A number of cross-sectional and experimental studies have reported that maternal khat chewing during pregnancy can adversely affect pregnancy outcomes. In a large-scale research by Eriksson involving 1141 Yemeni pregnant women, khat chewing pregnant women were give birth to more LBW neonates than non-consumers (48). Some Ethiopian researchers have also noted a link between maternal khat use during pregnancy and adverse pregnancy outcomes (49–54). An institution-based cross-sectional study conducted at selected health care facilities in Harar town showed that the odd of PROM was 1.5 times higher among khat consumers as compared to non-consumers (54). According to a study conducted in Eastern Ethiopia, women who chewed khat on a daily basis had a 29% higher risk of anemia than those who did so only sometimes or never (55).

### Global prevalence

Globally, over 10 million people are regular khat users (56). Recently, significant migration from East Africa has been connected to the expansion of khat usage to neighboring nations, Europe, and the rest of the globe (57). A study conducted in Yemen reported that the prevalence of khat use among adult women was 29.6% (58). Khat chewing is becoming an increasingly common habit among women in Ethiopia. According to 2016 Ethiopian Demographic Health Survey (EDHS), the nationwide prevalence of khat use was estimated to be 12% of women had experience of khat chewing (59). According to a secondary data analysis of the 2016 Ethiopia demographic and health survey, the prevalence of khat consumption among women of reproductive age was 8.4% (60). The prevalence of khat intake by pregnant women varies from 9.9% to 35.8% in Ethiopia (55, 61–65).

### Study gap

The majority of studies on khat use in women have been undertaken on subgroups of women, with the majority of studies being conducted on pregnant women (55, 61–65). Hence, the results of these studies cannot be generalized to all women of reproductive age. Furthermore, the effects of some potential factors, like stressful life events, knowledge about and attitude towards the effects of khat have been given little attention and are not well understood. Previous studies that examined consumption of khat in the Arabian Peninsula and Sub-Saharan Africa (55, 58, 60–65) including Ethiopia, focused primarily on individual-level factors with little attention to community-level factors.

Individual characteristics connect with community characteristics to which individuals belong, implying that women are impacted by their social surroundings, and that the features of those communities are influenced in turn by the individual factors that comprise that group. However, the majority of study findings (55, 58, 60–64) did not address how factors impact across the levels by using a multilevel analysis. As a result, all of these studies are subject to an atomistic or ecological fallacy.

Furthermore, the failure to identify community-level characteristics related to khat does not provide a full picture of the dimensions of khat usage, does not provide significant information to policymakers for action, and does not highlight a clear gap for future studies. In light of this, the aim of this study is to examine the individual and community-level factors associated with khat use among women of reproductive age by using multilevel modeling and to provide evidence for policymakers to fully understand potential factors influencing khat use.

## Methods

### Study design and setting

**Figure 1:**
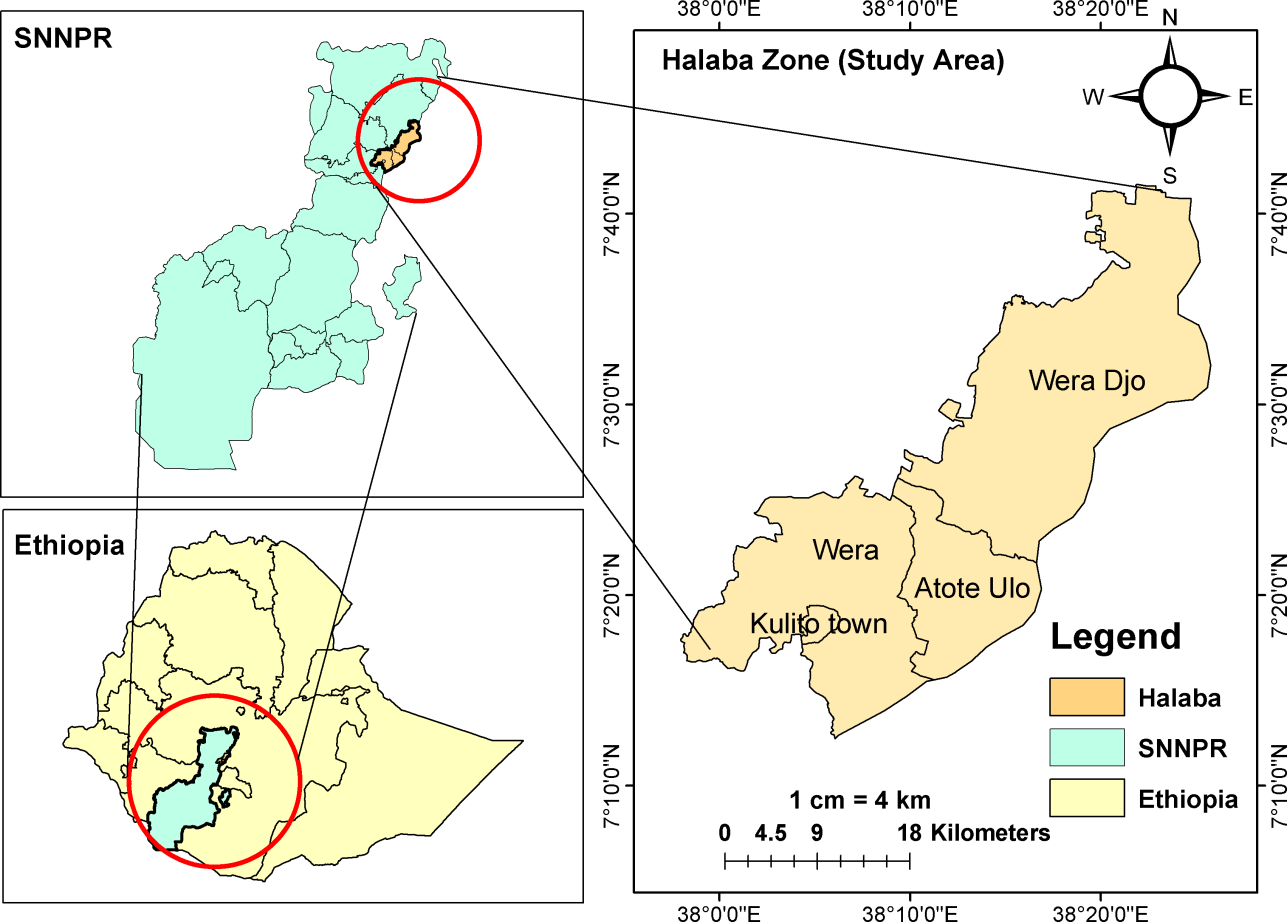
Maps showing Ethiopia, SNNPR and Halaba zone (the study area). Source: The shape file accessed from Humanitarian Data Exchange website (https://data.humdata.org/dataset/cod-ab-eth). The shape file is public domain and can be used freely.

A community-based cross-sectional study was conducted from February 20 to July 20, 2023 in the Halaba Zone, Southwest Ethiopia. Halaba zone is one of the 14 zones in the Southern Nation’s Nationalities and Peoples’ Region (SNNPR) of Ethiopia region (**Figure 1**). It is located in southwest Ethiopia, 315 kilometers from Addis Ababa, Ethiopia’s capital city. The Halaba zone is characterized by a dry climate, with approximately 14% low-land and 86% mid-land areas. It is structured into three districts and one town administration. The zone has 5 urban and 79 rural “kebeles” (the lowest administrative division in Ethiopia). According to the 2017 population projection value, this zone has a total population of 301,658 people, of which 151,545 were females and 244,582 (80.1%) were rural dwellers (66). Based on the zone agriculture office, the zone is well-known for cultivating khat, pepper, and pulses, which are valuable cash crops for farmers. The zone has one primary-level hospital, seven health centers, and fifty health posts.

### Source population and study population

All women of reproductive age who lived at the Halaba zone were the source population and all women of reproductive age who lived at the selected kebeles during the data collection periods were taken as a study population.

### Inclusion and exclusion criteria

#### Inclusion criteria

- All women of the reproductive age group (15-49) and being a resident of selected kebeles at least for 6 months were included in the study.

#### Exclusion criteria

- Women of the reproductive age group who are critically ill, unable to hear or talk during the study period were excluded from the study.

### Sample size determination and sampling technique

The sample size was determined by taking into account the outcome variable and different factors that were significantly associated with the outcome variable in previous studies, and the larger sample size was chosen to be used for this study. The sample size for the prevalence of khat chewing was computed by using single population proportion formula by assuming that the proportion of khat chewing among women was 8.4% (60), 95% confidence interval, 3% margin of error, a design effect of 2 and considering 10% of non-response rate. The calculated sample size was 722.

While a double population proportion formula was used to determine sample size for factors associated with khat chewing; assumption of a 95% confidence interval (CI) (2-sided), 80% power, 2.8% of outcome in the non-exposed category, 0.1% of outcome in the exposed category, and non-exposed to an exposed ratio of 1:1; and considering “religion” as a predictor factor to bring a difference in two population based on the study conducted in Ethiopia (60), and a design effect of 2. The final sample size was 1659 women of reproductive age after adjusting for non-response (10%). As a result, the sample size computed using the double population proportion formula was considered sufficient for determining the prevalence of khat chewing and its associated factors.

A multistage stratified sampling method was used to identify study subjects. Out of four districts in the zone, three were selected randomly. Then the selected districts were first stratified into rural and urban kebeles. Then 11 out of 44 rural kebeles and 1 out of 5 urban kebeles (25%) were randomly selected. Following that, the entire sample size was proportionally assigned to each selected kebele, and systematic random selection was employed to select households where eligible women were living. The first household was chosen by lottery method, while the following households were chosen by skipping k^th^-intervals. The sampling interval (k) of the households in each kebele was calculated by simply dividing the entire number of households to the assigned sample size. If there is more than one eligible woman in the home, the woman to be questioned was chosen by a lottery method. When no eligible woman was found in the chosen household, the next house in the right direction was visited.

### Study variables

#### Dependent variable

- The dependent variable is current khat chewing, which is operationalized as chewing khat within 30 days preceding the study. It is a binary variable categorized as ‘Yes’ or ‘No’ (59, 60).

#### Independent variables

- Nineteen independent variables made up of sixteen individual-level factors, and three community-level factors were considered in this study. These variables were selected by reviews of previous published literatures (55, 58–64). The individual-level factors are age of women, educational level, religion, marital status, occupational status, household wealth index, pregnancy status, breastfeeding status, number of living children, mass media exposure, ever heard the harmful effect of khat chewing, knowledge about the harmful effect of khat consumption, attitude towards khat chewing, social support, life-threating experience, and friend’s khat use.
- The community-level factors are residence, community women’s mass media use, and community wealth status. The aggregate community level factors were created by aggregating individual level characteristics at the cluster/kebele level and the aggregate variables were classified as high or low based on the proportion distribution or mean/median values determined for each community. A histogram was used to check the distribution of the aggregated values. If the aggregate variables were normally distributed, the mean score was used as the cut off point for categorization; otherwise, the median value was utilized. Community mass media exposure was classified as low if the percentage of women exposed to mass media in the community was <50% whereas high if the proportion was ≥50%. The median value of the wealth index was 2. Then the aggregated communities were classified into low if the median value of the community was below 2 and high if the median value of the community was greater than or equal to 2.

### Data collection tool, measurement and procedure

An interviewer-administered questionnaire was used for the data collection. The questionnaire has different elements, including socio-demographic and economic characteristics, reproductive characteristics, social support Scale, list of threating events, and questions on women’s attitudes and knowledge regarding khat chewing. The questionnaires also include questions regarding alcohol use, current khat use, age of initiation of khat use, frequency of khat use, time spent in khat session, source of khat, reason for khat use, close friends’ khat consumption and current attempt to reduce khat use.

### Wealth status

A standardized questionnaire adopted from the EDHS 2016 was used to assess households’ wealth status (59). Principal component analysis was used to measure socioeconomic status. Variables included house ownership, household assets, household features, and amenities. The household characteristics and facilities were dichotomized as “not improved” and “improved” and coded as “0” (unimproved) and “1” (improved). The wealth index was determined independently for urban and rural areas. The wealth index was weighted for urban and rural areas and then combined to produce a single wealth index. The wealth status was classified into three groups (poor, medium and rich).

### Social support

The Oslo-3 Social Support Scale (OSSS-3) (67) is a fast and easy tool for assessing the degree of social support. As a result, the level of social support was measured using the Oslo-3 item Social Support Scale, with a total score ranging from 3 to 14. The OSSS-3 consists of three questions that inquire about the number of close confidants, the sensation of care from others, and the connection with neighbors, with an emphasis on the availability of practical assistance. The sum scores were categorized as poor (3-8), moderate (9-11) and strong (12-14). The OSSS-3 has good predictive and convergent validity (68).

### Life threatening experience

The List of Threatening Experiences (LTE) (69) was used to examine stressful life experiences that occurred in the six months preceding the evaluation. The LTE includes 12 types of significant life events. Women who report “no” for all items were categorized as no trauma, one or more “yes” as expose to a trauma.

### Mass media exposure

We evaluated women’s mass media exposure as well. When women listen to the radio or watch television at least once a week, they were considered to be exposed to mass media; otherwise, they were not (59).

### Knowledge regarding the effects of khat

Knowledge of women regarding the health effects of khat was evaluated using a series of 18 questions adopted from different literatures (70, 71). The knowledge questionnaire was completed on a true/false basis with an extra “I don’t know” option. A correct response received three points, an unknown response received two points, and a wrong response received one point. The total score for knowledge varied from 0 to 54. Using the original Bloom’s cut-off point, the total level of knowledge was classified as good if the score is 80-100% (43-54 points), medium if the score is 60-79% (32-42 points), and low if the score is less than 60% (32 points). The Cronbach alpha for the knowledge questions was 0.94.

### Attitude towards khat chewing

Attitude towards khat chewing was assessed using 21 questions. Responses to attitude questions were graded on a 5-point Likert scale, ranging from 1 to 5 (1=strongly disagree, 2=disagree, 3=neutral/not sure, 4=agree, 5=strongly agree) (70, 71). The overall attitude of participants was classified as positive if the score is between 80 and 100% (84-105 points), neutral if the score is between 60 and 79% (63-83 points), and negative if the score is less than 60% (63 points). A positive attitude towards khat chewing means having a perception of that khat chewing has no negative effect on women health, fetus health, child care, family income, social interaction and it should not be avoid during pregnancy and lactation. The Cronbach alpha for the attitude questions was 0.93.

### Data management and analysis

#### Descriptive statistics

Double data entry and data cleaning procedures were performed using EPI-INFO version-7 statistical software and later exported to STATA version 14.0 for analysis. Using information from the literature, continuous variables were grouped, and categorical variables were reclassified accordingly. Individual-level characteristics were aggregated at the community (cluster) level to create the aggregate community-level explanatory factors. A proper descriptive statistical analysis of the data was conducted for the various individual and community-level characteristics of the women. Categorical variables were reported using frequency and percentage, while continuous explanatory factors were reported using mean and standard deviation (SD). Furthermore, cross-tabulation was made to show the proportion of each characteristic in relation to khat chewing.

#### Model building

During the analysis, women’s characteristics were regarded as individual-level (level-I) variables, while cluster characteristics were regarded as community-level (level-II) variables. To evaluate the effect of individual and community factors on khat chewing, a two-level model was developed in which individual reproductive age women (level I) are nested within the kebele/community (level II). During the analysis, bivariable two-level logistic regression was fitted first, and factors with a p-value ≤ 0.25 were regarded as candidate for multivariable analysis. The STATA syntax ‘melogit’ was used for the bi-variable and multivariable multilevel binary logistic regression analysis. Four models were fitted with variables of interest.

Model I (Null model): We began with a null model with only a random intercept. It was fitted to determine the total variance in the use of khat among childbearing age women in different communities and to justify the application of multilevel logistic analysis by calculating Intra-class Correlation Coefficient (ICC).

Model 2 (Only individual-level predictors): This model examined the effect of individual characteristics on khat use among women of reproductive age. This model includes individual-level independent variables that were statistically significant in the bi-variable multilevel analysis.

Model 3 (Only community-level predictors): It assessed the effects of community variables on khat use. This model includes community-level independent variables that were statistically significant in the bi-variable multilevel analysis.

Model 4 (Both individual and community level predictors): We examined the influence of both individual and community-level factors using this model at the same time.

The measures of association (fixed-effects) estimate the association between likelihood of khat chewing as the adjusted odd ratio (AOR) with 95% CI of various independent variables was expressed. At multivariable analysis independent variables with p-values ≤ 0.05 with confidence interval not including the null value (OR = 1) was considered as statistically significant variables with khat chewing.

#### Model fit statistics

The Akaike Information Criterion (AIC), Bayesian Information Criterion (BIC), and log-likelihood ratio (LLR) were used to determine the goodness of fit of the adjusted final model in comparison to the prior models. The model with the lowest values of BIC and AIC and the highest value of LLR was considered to be the best fit model.

#### Multicollinearity

By doing the pseudo linear regression analysis, the multicollinearity was checked and the Variance Inflation Factor (VIF) at a cut-off point of 10 (72). The findings indicated no evidence of high collinearity among the independent variables (Mean VIF=1.9, Minimum VIF=1.07, Maximum VIF=4.83).

#### Parameter estimation

The measures of variation (random-effects) for each model was reported using ICC, proportional change in variance (PCV) and Median Odds Ratio (MOR) to measure the variation of khat chewing across kebeles. The ICC shows the variation in khat use for women due to community characteristics. The higher the ICC (ICC > 5%), the more relevant is the community characteristics for understanding individual variation in khat chewing (73).

MOR is the median of a set of odds ratios calculated by comparing two respondents with identical individual-level characteristics from two randomly chosen, distinct clusters, i.e. difference in cluster random effect value (74).

PCV was calculated for each model with reference to the null model to see relative contribution of individual or/and community-level to explain variation in khat chewing.

#### Data quality management and control

The quality of the data was ensured through proper questionnaire design and pre-testing, as well as proper training of interviewers and supervisors on data the collection process. The data collection was pre-tested in one of the kebeles other than the selected study area, but has comparable socio-demographic features with the study population. As a result, it was done among 5% of the entire sample size, and necessary revisions were made to the questionnaire prior to data collection.

Supervisors and data collectors were trained on the data collection procedure for three days in order to have a common understanding. The questionnaire was reviewed and checked for completeness by the primary investigator and direct supervisors every day, and any necessary input was supplied to data collectors the next morning prior to data collection.

## Results

### Socio-demographic and reproductive characteristics of study participants

A total of 1573 study participants were interviewed, with a 95.0% response rate. The lack of response was owing to the participant’s lack of interest in participating and a lack of time. The mean age (SD) of the women was 29.08 ( ± 7.54) years, with the majority aged between 25 and 34 years. Majority of the study participants were ever married (92.5%) and followers of the Muslim religion (88.4%). Regarding educational level, more than two-thirds (74.9%) of women had no formal education, while 13.2% had completed secondary and above education. About 84.7% and 2.5% of women were housewives and government employees by occupation, respectively. Around 34.1% and 33.3% of respondents were from poor and rich households, respectively. With regard to the number of children a woman has, 85.8% of women have more than or equal to one child. Over three-quarters of the women were neither pregnant nor breastfeeding (**Table 1**).

**Table 1:** Socio-demographic and reproductive characteristics of women of reproductive age in Halaba Zone, South Ethiopia, 2023 (n=1573).

Individual level variables with their category	Total n (%)	Khat use				Chisq. *(p*-value)
		Yes n (%)		No n (%)		
Age (Year)						
15-24	337(21.4)	140(41.5)		197(58.5)		*p<*0.001
25-34	831(52.8)	573(68.9)		258(31.1)		
35 and above	405(25.8)	323(79.7)		82(20.3)		
Mean± SD	29.08 ± 7.54					
Marital status						
Never married	118(7.5)	28(23.7)		90(76.3)		*p*<0.001
Ever married	1455(92.5)	1008(69.3)		447(30.7)		
Level of education						
Unable to read and write	1179(74.9)	915(77.6)		264(22.4)		*p*<0.001
Primary school	187(11.9)	79(42.2)		108(57.8)		
Secondary school and above	207(13.2)	42(20.3)		165(79.7)		
Religion						
Muslim	1390(88.4)	1028(74.0)		362(26.0)		*p*<0.001
Orthodox	115(7.3)	8(7.0)		107(93.0)		
Protestant	68(4.3)	–		68(100)		
Occupation						
Student	91(5.8)	14(15.4)		77(84.6)		*p*<0.001
Day laborer	49(3.1)	17(34.7)		32(65.3)		
Government employee	40(2.5)	13(32.5)		27(67.5)		
Merchant	61(3.9)	20(32.8)		41(67.2)		
House Wife	1332(84.7)	972(73.0)		360(27.0)		
Household wealth status						
Poor	535(34.1)	376(70.3)		159(29.7)		*p*<0.05
Middle	512(32.6)	316(61.7)		196(38.3)		
Rich	522(33.3)	340(65.1)		182(34.9)		
Current pregnancy status						
Pregnant	143(9.1)	103(72.0)		40(28.0)		*p*>0.05
Not pregnant	1430(90.9)	939(65.7)		491(34.3)		
Lactating						
Yes	601(38.2)	393(65.4)		208(34.6)		*p*>0.05
No	972(61.8)	634(65.2)		338(34.8)		
Number of living children						
0	224(14.2)	83(37.1)		141(62.9)		*p*<0.001
≥1	1349(85.8)	958(71.0)		391(29.0)		

### Mass media exposure, women’s knowledge and attitude regarding khat use

**Figure 2:**
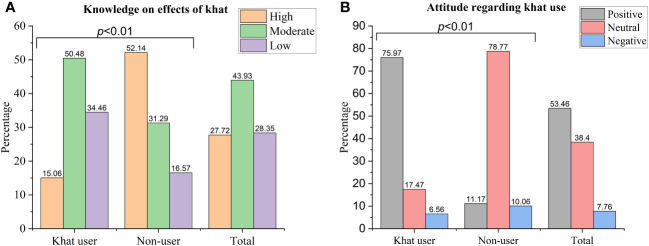
Women’s knowledge about and attitude towards khat use adverse health and social effects among women of reproductive age in Halaba Zone, South Ethiopia, 2023. **(A)** knowledge on effects of khat **(B)** Attitude regarding khat use.

Less than one-third (31.5%) of women had at least weekly access to any of the two mainstream media (television and radio). Five hundred eighty-two (37.0%) women heard about the health effects of khat from various sources. Regarding the main source of information for respondents, 75.9% heard it from other people. Furthermore, 69 (11.9%) obtained it via television, 39 (6.7%) from radio, and 32 (5.5%) from healthcare practitioners (**Table 2**). Regarding the overall knowledge of research participants, 27.7% had good knowledge regarding the impact of khat consumption based on nineteen knowledge items. Taking twenty-one attitude-evaluation questions into account, only 7.75% of the women had an unfavorable attitude and 53.5% had a positive attitude regarding khat consumption (**Table 2** and **Figure 2**).

**Table 2:** Mass media exposure and information regarding khat use risks among women of reproductive age in Halaba Zone, South Ethiopia, 2023.

Individual level variables with their category	Total n (%)	Khat use		Chisq. *(p*-value)
		Yes n(%)	No n(%)	
Watch television or listen to the radio at least once a week (n=1573)				
Yes	495(31.5)	158(31.9)	337(68.1)	*p*<0.001
No	1078(68.5)	878(81.4)	200(18.6)	
Ever heard the health risk of khat use (n=1573)				
Yes	582(37)	203(34.9)	379(65.1)	*p*<0.001
No	991(63)	833(84.1)	158(15.9)	
Source of information among heard (n=582)				
Radio	39(6.7)	–	–	
Television	69(11.9)	–	–	
Health professional	32(5.5)	–	–	
Other people	442(75.9)	–	–	

### Social support and life threatening experience of study participants

Regarding the finding on social support measured by the OSSS-3 scale, 34.3%, 30.5%, and 35.2% scored poor, moderate, and strong social support, respectively. Major stressful events experienced in the previous six months were not common and were reported by 14.4% of the respondents (**Table 3**).

**Table 3:** Psychosocial characteristics of women and friend khat use among women of reproductive age in Halaba Zone, South Ethiopia, 2023 (n=1573).

Individual level variables with their category	Total n (%)	Khat use		Chisq. *(p*-value)
		Yes (%)	No (%)	
Social support				
Poor	540(34.3)	396(73.3)	144(26.7)	*p*<0.001
Medium	479(30.5)	322(67.2)	157(32.8)	
Good	554(35.2)	318(66.3)	236(33.7)	
Life threating experience in the previous 6 months				
None	1346(85.6)	863(64.1)	483(35.9)	*p*<0.001
One and above	227(14.4)	173(76.2)	54(23.8)	

### Community-level characteristics of study participants

Over three-fourths (76.5%) of the respondents were rural residents. The majority (70.2%) of the respondents were from communities with lower level of mass media use. More than nine in ten of the respondents (92.6%) were from communities with a low percentage of poverty (**Table 4**).

**Table 4:** Community-level characteristics of study participants, Halaba Zone, South Ethiopia, 2023 (n=1573).

Community level variables with their category	Total n (%)	Khat use		Chisq. *(p*-value)
		Yes (%)	No (%)	
Place of residence				
Rural	1203(76.5)	956(79.5)	247(20.5)	*p*<0.001
Urban	370(23.5)	80(21.6)	290(78.4)	
Community-level women’s mass media use				
Low	1105(70.2)	881(79.7)	224(20.3)	*p*<0.001
High	468(29.8)	155(33.1)	313(66.9)	
Community-level women’s wealth status				
Low	117(7.4)	80(68.4)	37(31.6)	*p*<0.001
High	1456(92.6)	956(65.7)	500(34.3)	

### Khat use pattern and related characteristics

The pattern of khat chewing among women of reproductive age is shown in **Table 5**. Out of the 1573 women, 1042 (66.2%, 95%CI: 63.9-68.6) had ever chewed khat while 1036 (65.9%, 95%CI: 63.5-68.2) had chewed khat within 30 days preceding the date of the interview. The average age of initiation to khat use for chewers was 17.4 ( ± 1.20) years. More than half of the women (63.2%) started chewing at the age of 18 years or above, but for 36.8% of respondents (n=378) it was younger than eighteen years old. Of current chewers, 80.0% chewed 1 bundle per session, 11.3% chewed half, and 0.8% chewed two bundles. Among current khat chewers, 21.9% chewed it for 1-6 days, 1.5% for 7-14 days, and 76.5% for 15 days and more. Women chewed khat on an average ( ± SD) of 23 ( ± 11.4) days in the previous 30 days.

**Table 5:** Khat use pattern and related parameters among respondents, Halaba Zone, South Ethiopia, 2023.

Characteristics	Frequency	Percentage	
Ever chewed khat (n=1573)			
Yes	1042	66.2	
No	531	33.8	
Khat use in the previous 30 days (n=1573)			
Yes	1036	65.9	
No	537	34.1	
Age of khat initiation (n=1036)			
Less than 18 years	378	36.8	
18 years or more	658	63.2	
Mean± SD	17.38 ± 1.2		
Number of days used khat in the previous 30 days (n=1036)			
1-6 days	227	21.9	
7-14 days	16	1.5	
≥ 15 days	793	76.6	
Mean± SD	23 ± 11.4		
Time spent in khat sessions (hours) (n=1024)			
One hour	3	0.3	
2-4 hours	346	33.8	
5 and more hours	675	65.9	
Mean± SD	5.61 ± 1.79		
Amount of khat chewed during typical khat session (bundle) (n=977)			
One fourth	77	7.9	
Half	110	11.3	
One	782	80.0	
Two	8	0.8	
Substance used with khat (n=1036)* khat users			
Sugar	912	88.0	
Water	266	25.7	
Ground nut	97	9.4	
Coffee	50	4.8	
Soft drink	11	1.1	
Common source of khat (n=1036)			
From sellers	123		11.9
From other people	143		13.8
From family member	770		74.3
Number of friends that use khat (n=1557)			
None of them	442	28.4	
Some of them	362	23.2	
Most of them	340	21.8	
All of them	413	26.6	
With whom do you commonly chew khat (n=1036)			
Alone	84	8.1	
Friend	123	11.9	
Husband	778	75.2	
Relative	51	4.8	
Willing to quit khat use (n=1036)			
Yes	75	7.2	
No	884	85.4	
No response	77	7.4	
Alcohol use in the previous 30 days (n=1573)			
Yes	24	1.5	
No	1549	98.5	

* khat users.

Each khat session lasted at least 5 hours for over two-thirds (65.9%) of the women. The mean number of hours spent on khat sessions per day was 5.61( ± 1.79) hours. In terms of items consumed with khat, the majority of research participants chewed khat along with sugar (88.3%), whereas 1.1% consumed soft drinks. When women were asked how simple it was to get khat, the majority said it was somewhat easy. From those who chew khat, 13.8% declared that they were supplied with khat from other people, 11.9% bought khat themselves, and 74.3% of participants get khat at home because other residents chew. Twenty-six percent of women considered that all their friends used khat. More than three-fourths (85.3%) of the khat users were not willing to quit khat (**Table 5**).

**Figure 3:**
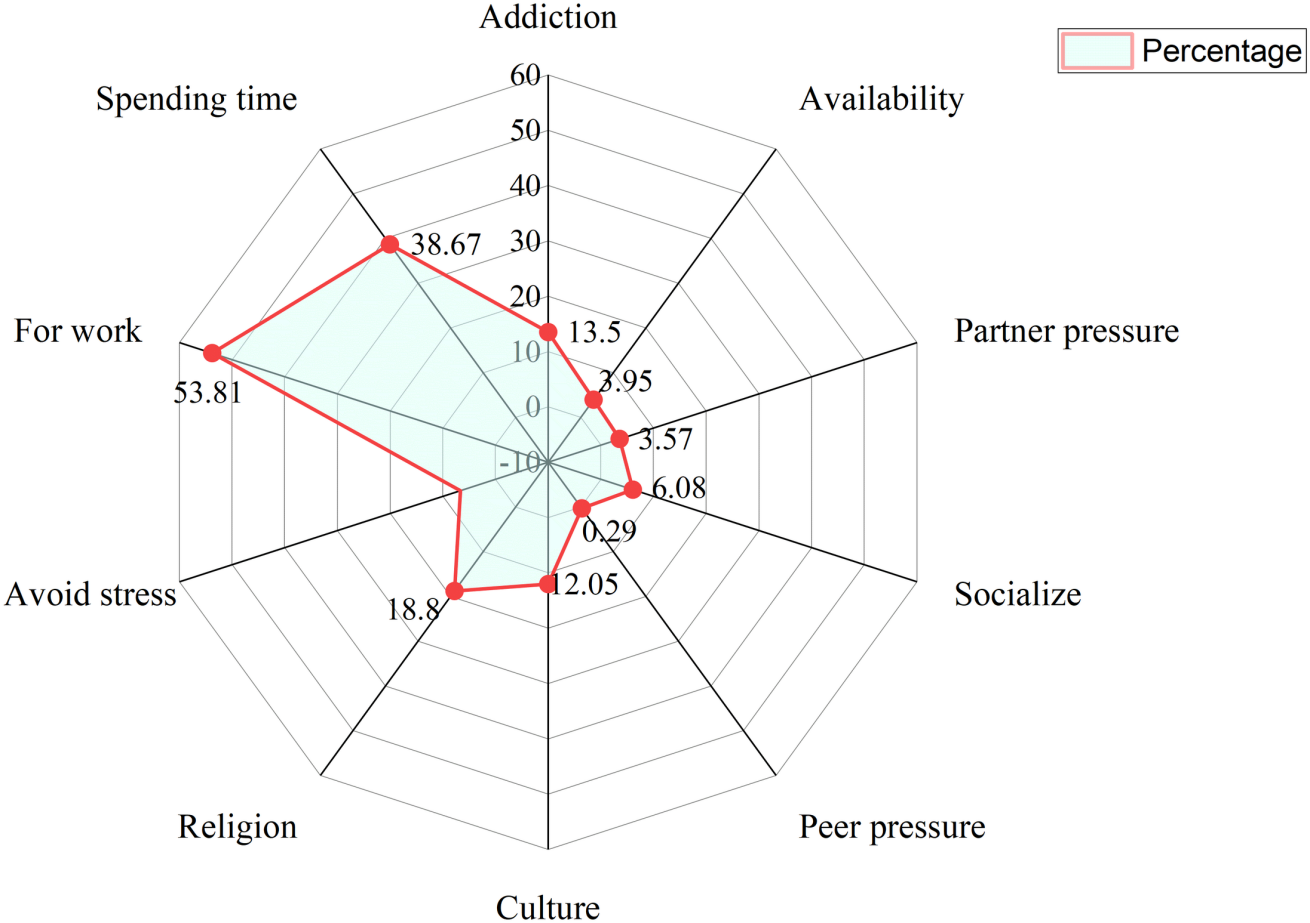
Reasons for khat use among women of reproductive age in Halaba zone, South Ethiopia.

A number of factors were identified as reasons for khat use among women of reproductive age. Using khat for performing daily work and for spending time were the main reasons for the initiation of khat use among khat users in the study population, which accounts for 53.8% and 38.7% of the cases, respectively. One hundred ninety-five (18.8%) reported chewing khat because they used it to perform religious practices. One hundred forty (13.5%) reported chewing khat because they were already addicted to khat. Other reasons for khat usage indicated by participants included cultural acceptance, improving social interaction, peer pressure, partner pressure, to relieve life stressors, and the easy availability of khat (**Figure 3**).

### Multilevel mixed-effects analysis

#### Model fit statistics

We conducted a two-level mixed-effects multivariable logistic regression that is aimed at identifying individual-level and community-level factors of khat use among women of reproductive age in Halaba zone. Those four different models were built to analyze factors accordingly. The fixed effects and the random intercepts for the use of khat are presented in **Table 6**. In model I (empty model), the ICC indicated that 18.67% of the total variability for khat use was due to differences between kebeles while the remaining unexplained 81.33% of the total variability of khat use was attributable to individual differences.

**Table 6:** Multilevel mixed effect analysis of individual and community level factors associated with khat use among women of reproductive age in Halaba zone, South Ethiopia, 2023.

Variables with their category	Model 1 (Empty model)	Model 2 AOR (95%CI)	Model 3 AOR (95%CI)	Model 4 AOR (95%CI)
Age				
15-24^a^		1		1
25-34		3.78(2.42, 5.91)^***^		4.01(2.56, 6.28^)***^
35 and above		5.62(3.23, 9.79)^***^		6.35(3.62, 11.13)^***^
Marital status				
Never married ^a^		1		1
Ever married		2.70(1.20, 6.05)^*^		2.41(1.10, 5.31)^*^
Level of education				
Can’t read and write ^a^		1		1
Primary school		0.96(0.58, 1.59)		1.08(0.65, 1.80)
Secondary school and above		0.26(0.14, 0.47)^***^		0.28(0.16, 0.51)^***^
Wealth status				
Poor ^a^		1		1
Middle		1.84(1.21, 2.80)^**^		1.92(1.25, 2.93)^**^
Rich		1.89(1.21, 2.97)^**^		1.75(1.12, 2.75)^*^
Mass media use				
Yes ^a^		1		1
No		3.09(1.93, 4.96)^***^		2.98(1.85, 4.81)^***^
Heard the risk of khat use				
Yes ^a^		1		1
No		2.13(1.36, 3.35)^**^		2.04(1.30, 3.20)^**^
Knowledge regarding khat use risks				
High ^a^		1		1
Moderate		1.81(1.21, 2.71)^**^		1.78(1.19, 2.67)^**^
Low		2.85(1.70, 4.77)^***^		3.12(1.85, 5.24)^***^
Attitude regarding khat use risks				
Negative ^a^		1		1
Neutral		1.16(0.72, 1.86)		1.20(0.74, 1.93)
Positive		13.31(7.84, 22.59)^***^		11.55(6.76, 19.71)^***^
Social support				
Poor ^a^		1		1
Moderate		0.53(0.35, 0.82)^**^		0.53(0.35, 0.82)^**^
Strong		0.43(0.28, 0.64)^***^		0.43(0.28, 0.64)^***^
Friends’ khat use				
All of them ^a^		1		1
Most of them		0.77(0.46, 1.27)		0.79(0.48, 1.31)
Some of them		1.06(0.65, 1.74)		1.10(0.67, 1.80)
None of them		0.32(0.21, 0.50)^***^		0.31(0.20, 0.48)^***^
Residence				
Urban ^a^			1	1
Rural			11.95(5.16,27.69)^***^	5.06(1.82, 14.09)^**^
Community mass media use				
Low ^a^			1	1
High			0.75(0.37, 1.51)	1.90(0.78, 4.60)
Community women wealth				
Low ^a^			1	1
High			2.01(1.05, 3.83)^*^	1.81(0.89, 3.68)
Random effect results				
Community-level variance	0.75	0.18	0.05	0.03
ICC (%)	18.67	5.13	1.65	0.87
MOR (95%CI)	2.28	1.50	1.24	1.18
PCV (%)	Reference	76.00	93.00	96.00
Model fit statistics				
Log-likelihood	-808.32	-485.80	-797.64	-481.65
AIC	1620.63	1011.61	1605.29	1009.31
BIC	1631.36	1118.77	1632.09	1132.55

^a^ is a reference category; 95%CI is the 95% confidence interval; Significant at *p ≤ 0.05, ** p<0.01, ***p<0.001; AOR is adjusted odds ratio; ICC is the Inter-Cluster Correlation; MOR is Median Odds Ratio; PCV is the Proportional Change in Variance; AIC is Akaike’s Information Criterion; BIC is Bayesian Information Criterion.

Additionally, the MOR was 2.28 in the empty model, which indicated that there was variation in khat use between kebeles. If we randomly select two women from different kebeles, if we transfer women from low khat use kebeles to higher khat use clusters, she could have 2.28 times higher odds of khat use. The ICC value declined across the individual factors, community factors and combined level factors model with a value of 5.13, 1.65 and 0.87%, respectively. In another way, the PCV findings indicated that the predictor variables to the null model better explained the factors associated with khat use. The PCV value for model 2 was 76%, for model 3 was 93% and model 4 was 96%. Model 4 (combined individual-level and community-level factors) indicated that 96% of the community-level difference on khat use was described by the combined factors at both the individual and community levels.

The AIC and BIC values showed a successive reduction, which means a substantial improvement in each of the models over the previous model. Model 4, which included all the individual and community-level factors had the lowest AIC of 1009.31 and the highest log-likelihood ratio of -484.65. Therefore, the final model (Model 4) was the best fit model for predicting the occurrence of khat use among women of reproductive age. The finding was reported based on model 4. In Model 4, individual-level factors (such as women’s age, current marital status, educational level, wealth status, mass media access, heard the risk of khat use, knowledge regarding khat use risks, attitude towards khat use, social support, and friend’s khat use) and community-level factors (such as place of residence) were significantly associated with khat use among women of reproductive age (**Table 6**).

### Individual and community-level factors associated with khat use

After adjusting for individual and community-level factors, the odds of khat use were 4 times (AOR =4.01; 95%CI: 2.56-6.28) higher among those aged 25-34 years and 6 times (AOR =6.35; 95%CI: 3.62-11.13) higher among those aged 35 years and above compared with women aged 15-24 years. Looking at educational level, women who had secondary education and above were 72% (AOR=0.28; 95%CI: 0.16-0.51) less likely to use khat as compared to women who had no education. Women from wealthier households were more likely to use khat than those from the poorest households. Specifically, the odds of khat use were higher for women in the middle (AOR=1.92; 95%CI: 1.25-2.93), and richest (AOR=1.75; 95%CI: 1.12-2.75) households, as compared to the poorest.

The likelihood of khat use was significantly affected by access to mass media. When compared to women who had mass media exposure, women who had no exposure to it were nearly three folds more likely to use khat (AOR=2.98; 95%CI: 1.85-4.81). On the other hand, women who did not hear about the adverse effects of khat were more likely to use khat (AOR = 2.04; 95%CI: 1.30, 3.29) compared to women who hear about it. Women who had low and moderate knowledge about effects of khat use were almost three (AOR =3.12; 95%CI: 1.85-5.24) and two (AOR =1.78; 95%CI: 1.19, 2.67) times more likely to use khat as compared to women who had good knowledge, respectively.

Women who had positive attitudes towards khat use were eleven (AOR =11.55; 95%CI: 6.76-19.71) times more likely to use khat as compared to women who had a negative attitude. Regarding social support, women who had strong social support were 57% less likely (AOR =0.43; 95%CI: 0.28-0.64) to use khat compared to women who had poor social support. When none of their friends used khat, their own chance of chewing khat was 69% less likely (AOR=0.31; 95%CI: 0.20-0.48) compared with when all of their friends used khat. After holding other variables constant, women living in rural kebeles were almost 5.06 times more likely (AOR =5.06 95%CI: 1.82-14.09) to use khat compared to their urban counterparts (**Table 6**).

## Discussion

This study aimed to examine the prevalence and multilevel factors associated with khat use among women of reproductive age in Halaba zone, South Ethiopia. To the best of our knowledge, this is the first study to examine the role of individual and community level factors on khat consumption using multilevel mixed effects analysis. Identifying these factors is important to take interventions for the problem and improve women’s health in the area.

According to the findings of this study, khat use was found to be a significant public health problem in Halaba zone. This study revealed that the prevalence of khat use among women of reproductive age was 65.86% (95%CI: 63.50, 68.20). Regarding prevalence, our result is higher than studies carried out in Yemen (29.6%) (58), Ethiopia (national study) (8.4%) (60), Eastern Ethiopia (34.62%) (55), and Southern Ethiopia (9.89%) (63). This could be explained by variations in social and cultural backgrounds as well as differences in the study population between the current study and previous studies. The prior two studies were conducted on pregnant women (55, 63). Pregnant women report lower rates of illicit drug usage than non-pregnant women because they are more concerned about their baby’s health throughout pregnancy.

The finding is also higher than studies conducted among the general population in rural Kenya (36.8%) (75), urban Kenya (10.7%) (76), and Uganda (10.5%) (77). The variation might be attributed to differences in khat legislation and khat availability. In Ethiopia, the production of khat has increased dramatically during the previous two decades, making the country the world’s biggest source. Currently, khat is one of Ethiopia’s largest crops by area of cultivation, the country’s second-largest export earner, and an important means of income for millions of Ethiopian farmers (78). Regarding legislation, Ethiopian law has little to say on khat. The Ethiopian government has prohibited khat chewing in academic institutions and workplaces (79). This is the only effort made by the government to decrease the consumption of khat in the country. As a result, consumption has extended from Ethiopia’s historical khat heartlands in the east and south to the majority of large areas (15). On the contrary, the Ugandan government approved the Narcotic Drugs Act, which categorized khat as a narcotic and banned its production and usage (80).

In this study, participants reported chewing khat on an average of 23 days in the previous 30 days. According to the 2016 EDHS, women chewed khat on average of 14.2 days over the past 30 days (60). This disparity could be explained by differences in the study area.

In this study, both individual-level factors (age, current marital status, level of education, wealth status, mass media exposure, heard the risks of khat use, knowledge about the effects of khat use, attitude towards khat use, social support, and friends’ khat use) and community-level factors (residence) were significantly associated with khat use. As shown in this study, the age of women was positively associated with khat use. Possibly, as the age of the women increases the practice of khat chewing increases which is consistent with the studies in Ethiopia (60, 65). Likewise, studies conducted in Yemen found that older women were more likely to use khat than younger ones (81). This finding might be justified by the fact that older women are vulnerable to psychiatric symptoms such as stress, anxiety, depression, loneliness, grief, and social isolation (82). Women may take khat as a copying strategy to reduce stress. An additional explanation might be that younger women are more likely to be under family authority, lowering their chance of exposure to khat use.

Higher education was significantly associated with decreased risks of khat use; this result was consistent with previous findings. Hence, education may increase women’s understanding of the negative effects of khat use and may increase women’s capacity to regulate chewing by quitting or reducing intake. Regarding wealth status, women from the rich wealth index were almost two times more likely to use khat compared to women from the poor wealth index. This finding is similar to studies conducted in Ethiopia and Yemen (58, 60) which indicate the likelihood of khat use was higher for women with rich wealth index as compared to women with poor wealth index. This might be because women in the rich wealth index quantile can afford the price of khat, which might increase the risk of khat use. In contrast to this, a study conducted in Yemen has shown that women with the poor wealth index were more likely to use khat as compared to women with rich wealth index quantile (81).

This study also revealed that the risk of khat use was lower among women who had strong social support as compared to their counterparts. The finding of this study was supported by studies done in different parts of Ethiopia (83, 84). One possible explanation for this finding is that a lack of social support can lead to psychological discomfort, feelings of loneliness, helplessness, and a perception of being disadvantaged. In turn, women use khat or other substances to alleviate such negative feelings. Furthermore, social capital theory states that social networks and linkages have an impact on health. Individuals with higher levels of social support and community cohesiveness are assumed to be healthier in general because they have better access to basic health information and health services (85).

The findings also show that having khat-chewing friends was a highly strong predictor of khat use among women of reproductive age. This is consistent with the literature’s results on peer pressure, the effect of social groupings, and the susceptibility of women to khat usage and other drugs. As a result, more awareness initiatives focusing on social groups and peers are required.

In this cross-sectional study, women who had no exposure to mass media had higher odds of khat use. This may be justified by the fact that exposure to various mass media can encourage women to utilize health services, and it also offers improved awareness and understanding, as well as improvements in attitudes, social expectations, and behaviors that can contribute to beneficial effects for health. We found that women who had poor knowledge of khat associated health risks and social problems were three folds more likely to use khat compared to women who had good knowledge. Knowing the dangers of khat use is essential for chewing cessation and prevention. Based on the knowledge-attitude-practice model (86), change in behavior involves obtaining relevant knowledge, changing related attitudes and, lastly, altering practices.

Furthermore, attitude regarding khat use was another important factor that associated with khat use. Women who had a positive were eleven times more likely to use khat compared to women who had a negative attitude, respectively. This could be because participants’ attitude regarding khat use was strongly influenced by their knowledge of the adverse effects of khat use. It is possible that as their knowledge improves, women will become more unfavorable towards khat use. Results of the study also found that women who reside in rural area were five times more likely to use khat than urban women. A possible reason that could explain why rural women are at higher risk of khat use is related to availability of khat. The perceived and real availability of substances from formal and informal sources can both impact the prevalence of drug use and related issues (87, 88). According to the Halaba zone agricultural office report, the majority of farmers in rural areas plant khat and engage in khat trading. As a result, rural women may readily obtain khat from their own or a neighboring farm.

### Limitations of the study

The current study’s findings should be considered in light of the following limitations. First, women’s khat chewing status was self-reported, with no biochemical confirmation. As a result, less socially acceptable behaviors may be underreported. Second, this study employed cross-sectional research methods, which can only demonstrate associations rather than causal linkages between the various independent factors and the outcome variable. Third, while the study included both rural and urban areas, it was not performed in a comparable manner. Fourth, only quantitative approaches were used to investigate the factors linked to khat consumption.

## Conclusions

Khat use among women of reproductive age was found to be very high in the study area. Individual and community-level characteristics were revealed to be significant determinants of khat use among women. Older women, ever-married, high wealth status, no media exposure, low knowledge about khat health risks, positive attitude towards khat use, and rural residence were positively associated factors of khat use. However, higher education level, strong social support, and non-user friends were protective factors of khat use among women of reproductive age. Therefore, policymakers and healthcare professionals should consider these factors identified in this study when planning and developing intervention programs to lessen the khat use among women of reproductive age. To reduce the burden, mass media campaigns and point-of-sale warnings in local languages intended for women of reproductive age are required. Additionally, khat use screening for all women of childbearing age, as well as referral to substance use disorder centers for those women identified as having khat use disorder, should become a standard of care in all health facilities. Future research needs to look into the effects of khat on maternal nutritional status, quality of life, and birth outcomes.

## Data availability statement

The original contributions presented in the study are included in the article/supplementary material. Further inquiries can be directed to the corresponding author.

## Ethics statement

The studies involving humans were approved by Institutional Research Ethics Review Committee of Wolaita Sodo University. The studies were conducted in accordance with the local legislation and institutional requirements. Written informed consent for participation in this study was provided by the participants’ legal guardians/next of kin.

## Author contributions

BW: Conceptualization, Data curation, Formal analysis, Investigation, Methodology, Project administration, Resources, Software, Supervision, Validation, Visualization, Writing – original draft, Writing – review & editing. TD: Conceptualization, Data curation, Formal analysis, Investigation, Methodology, Project administration, Resources, Software, Supervision, Validation, Visualization, Writing – original draft, Writing – review & editing. EW: Conceptualization, Data curation, Formal analysis, Investigation, Methodology, Resources, Software, Supervision, Validation, Visualization, Writing – original draft, Writing – review & editing. MA: Conceptualization, Data curation, Formal analysis, Investigation, Methodology, Resources, Software, Supervision, Validation, Visualization, Writing – original draft, Writing – review & editing. KD: Conceptualization, Data curation, Formal analysis, Investigation, Methodology, Software, Supervision, Validation, Visualization, Writing – original draft, Writing – review & editing.
